# Patient and Public Involvement in Technology-Related Dementia Research: Scoping Review

**DOI:** 10.2196/48292

**Published:** 2024-03-04

**Authors:** Pippa Kirby, Helen Lai, Sophie Horrocks, Matthew Harrison, Danielle Wilson, Sarah Daniels, Rafael A Calvo, David J Sharp, Caroline M Alexander

**Affiliations:** 1 Department of Therapies, Imperial College Healthcare NHS Trust London United Kingdom; 2 UK Dementia Research Institute Care Research and Technology Centre (UK DRI CR&T) London United Kingdom; 3 Department of Brain Sciences Faculty of Medicine Imperial College London London United Kingdom; 4 Helix Centre Institute of Global Health Innovation Imperial College London London United Kingdom; 5 Dyson School of Design Engineering Imperial College London London United Kingdom; 6 Department of Surgery and Cancer Imperial College London London United Kingdom

**Keywords:** dementia, technology, patient and public involvement and engagement, co-design, coproduction

## Abstract

**Background:**

Technology-related research on people with dementia and their carers often aims to enable people to remain living at home for longer and prevent unnecessary hospital admissions. To develop person-centered, effective, and ethical research, patient and public involvement (PPI) is necessary, although it may be perceived as more difficult with this cohort. With recent and rapid expansions in health and care–related technology, this review explored how and with what impact collaborations between researchers and stakeholders such as people with dementia and their carers have taken place.

**Objective:**

This review aims to describe approaches to PPI used to date in technology-related dementia research, along with the barriers and facilitators and impact of PPI in this area.

**Methods:**

A scoping review of literature related to dementia, technology, and PPI was conducted using MEDLINE, PsycINFO, Embase, and CINAHL. Papers were screened for inclusion by 2 authors. Data were then extracted using a predesigned data extraction table by the same 2 authors. A third author supported the resolution of any conflicts at each stage. Barriers to and facilitators of undertaking PPI were then examined and themed.

**Results:**

The search yielded 1694 papers, with 31 (1.83%) being analyzed after screening. Most (21/31, 68%) did not make clear distinctions between activities undertaken as PPI and those undertaken by research participants, and as such, their involvement did not fit easily into the National Institute for Health and Care Research definition of PPI. Most of this mixed involvement focused on reviewing or evaluating technology prototypes. A range of approaches were described, most typically using focus groups or co-design workshops. In total, 29% (9/31) described involvement at multiple stages throughout the research cycle, sometimes with evidence of sharing decision-making power. Some (23/31, 74%) commented on barriers to or facilitators of effective PPI. The challenges identified often regarded issues of working with people with significant cognitive impairments and pressures on time and resources. Where reported, the impact of PPI was largely reported as positive, including the experiences for patient and public partners, the impact on research quality, and the learning experience it provided for researchers. Only 4 (13%) papers used formal methods for evaluating impact.

**Conclusions:**

Researchers often involve people with dementia and other stakeholders in technology research. At present, involvement is often limited in scope despite aspirations for high levels of involvement and partnership working. Involving people with dementia, their carers, and other stakeholders can have a positive impact on research, patient and public partners, and researchers. Wider reporting of methods and facilitative strategies along with more formalized methods for recording and reporting on meaningful impact would be helpful so that all those involved—researchers, patients, and other stakeholders—can learn how we can best conduct research together.

## Introduction

### Background

Worldwide incidence of dementia is increasing. In the United Kingdom alone, there are approximately 1 million people living with dementia, with this figure expected to double by 2050 [1]. The total cost of care for people with dementia in the United Kingdom in 2019 was £34.7 billion (US $44.1 billion), with an expected increase to approximately £94 billion (US $119.5 billion) by 2040 [2]. Technology is increasingly cited as a means of supporting people with dementia and their formal and informal carers and reducing some of this economic burden. “Digitally enabled care” is a core component of the National Health Service Long Term Plan [3]—it is felt that technology has the potential to facilitate aging in place and reduce unplanned hospital admissions, with consequent economic benefits as well as improved health outcomes and quality of life [4,5]. Smart home systems, assistive technology devices, and other technologies are being developed with aims including supporting safety in people’s homes; enabling early detection of deterioration or ill health; supporting activities of daily living; and facilitating access to treatment, leisure activities, or social participation [5-8].

Despite rapid advancements in technology, the implementation of health and care–related technology for people with dementia has been slow, and there is increasing recognition of the many challenges in this area [9-11]. These challenges include ethical issues regarding privacy, autonomy, safety, and trust and the risk of creating or exacerbating health-related bias and inequality [12-16]. Researchers and technology developers must also understand the complex and changing needs of individual circumstances—there is a need for research to center on the person and their support network rather than on the technology itself if it is to be successful [17]. Therefore, understanding users’ perspectives is fundamental if we are to develop technologies that are acceptable, effective, and ethical [5,10,18,19]. One way to achieve this is through patient and public involvement (PPI).

PPI describes a partnership between patients, the public, and researchers in the research process itself. It is often described as research conducted “with” or “by” service users rather than research “about” or “for” them [20]. In addition to being seen as an ethical imperative, PPI aims to improve the efficiency and value of health research, recognizing that those with lived experience of health conditions or services will bring knowledge and experience that may increase the relevance of studies, improve recruitment and retention of participants, and improve dissemination of research findings [20-22]. PPI is now seen as an essential part of health and social care research—the Health Research Authority strongly advises PPI because of its likelihood of improving research quality and addressing the Research Ethics Committee’s key considerations [23]. Stakeholder engagement is a key part of the guidance from the International Council for Harmonisation of Technical Requirements for Pharmaceuticals for Human Use [24], and the National Institute for Health and Care Research (NIHR) makes it a condition of research funding [21]. The NIHR describes different approaches to involvement with increasing levels of power and influence for members of the public, from consultation (least power) to coproduction and user controlled (most power) [20].

PPI in dementia-related research has been gathering pace in recent years. Historically focusing more on the involvement of carers or other stakeholders, this has changed with more studies involving people with dementia themselves [25,26]. It is now well established that this supports and promotes a person-centered model of health care [27-31]. PPI should be conducted in a manner that promotes equality, diversity, and inclusion [20]. The NIHR emphasizes the need for researchers to enable the involvement of underrepresented groups and adapt their PPI approaches and activities to ensure accessibility for all groups affected by the project [32]. When planning and carrying out PPI in dementia research, this means the consideration of all groups affected by aging and dementia from diverse ethnic, racial, linguistic, geographic, and socioeconomic backgrounds.

The principle of stakeholder involvement is not unique to PPI. To understand the principles of terms such as “co-design” and “coproduction” within PPI, it is important to appreciate the context in which these terms have developed beyond just the health care sector. Within technology innovation, there has been a steady and increasing emphasis over the past 50 years on ensuring that a “human-centered” approach is taken to developing a new product or service [33]. Human-centered design emphasizes the need for fostering deep empathy with the people one is designing with, bringing end users into the design process as early as possible. Co-design can be a method of human-centered design. Co-design also stems from the 1970s, from a Scandinavian movement of participatory design, in which scientists, technologists, and design researchers acknowledged that “the people destined to use the system [must] play a critical role in designing it” [34]. Wider adoption of these human-centered design approaches has been seen in the last 15 years with methodologies such as the Design Council’s “Double Diamond” [35] helping visualize this iterative approach to innovation and widen adoption across nondesigners.

Considering the context of technology within health and social care, it is not surprising that practitioners from health and social care, design, and technology research have found themselves discussing what best practice should look like and what approaches or methods might facilitate meaningful innovation [36]. Regardless of the background, researchers across these disciplines agree on the need to move from a patient-centered or user-centered approach to a “co-production” approach in which users not only are observed or consulted but also work jointly as partners, with mutual respect and understanding of each other’s different knowledge and experiences and the contributions they can make [21,37,38]. The NIHR outlines 5 key principles of coproduction as part of a research project (Textbox 1).

**National Institute for Health and Care Research “Guidance on co-producing a research project”—key principles.**

1. Sharing of power—the research is jointly owned and people work together to achieve a joint understanding
2. Including all perspectives and skills—making sure the research team includes all those who can make a contribution
3. Respecting and valuing the knowledge of all those working together on the research—everyone is of equal importance
4. Reciprocity—everyone benefits from working together
5. Building and maintaining relationships—an emphasis on relationships is key to sharing power” [32]

Despite the recognition of the value of PPI and the recommendation of coproduction approaches [10,19], patient or other stakeholder involvement in technology-related dementia research is known to be variable in breadth and depth and sometimes absent altogether [11]. Older reviews show that the involvement of people with dementia has usually been as passive participants to be observed or at most as a group to consult but without any sharing of decision-making power [19,39,40]. A review of the literature published between 2011 and 2017 by Suijkerbuijk et al [41] demonstrated that, although there has been an increase in the involvement of people with dementia in technology research, reporting on the methods, barriers, facilitators, and impact remains minimal, making progress in this field challenging. This mirrors issues with PPI reporting (especially of impact) in the wider field of dementia research [25,42,43]. Given the increased attention that PPI has received in recent years as well as the rapid advances in technology-related health research, we anticipated that there would be many more papers published in the period from 2017 to 2022 worthy of review. In addition, the review by Suijkerbuijk et al [41] included papers with a broad range of methodologies to cover the concept of “involvement,” including the involvement of people with dementia as participants in qualitative research. To our knowledge, no review to date has explored the specific concept of PPI in technology-related dementia research.

### Objectives

Therefore, the objectives of this scoping review were as follows:

1. To describe the approaches to PPI used to date in technology-related dementia research, exploring who is involved, when, and how,
2. To describe the reported barriers to and facilitators of effective PPI in this area, and
3. To examine and report on the impact of PPI in this area.

## Methods

### Review Type

To gather the available literature in this area, a scoping review was conducted. Scoping reviews are often used in preference to systematic reviews in cases in which the body of literature is likely to be large and heterogeneous and to answer broad questions (such as “what is known about this concept?”) [44]. They are a useful way to map out the evidence, as opposed to systematic reviews, which often bring together literature on a particular subject with a more defined question, for example, about the efficacy of interventions [45]. The PRISMA-ScR (Preferred Reporting Items for Systematic Reviews and Meta-Analyses Extension for Scoping Reviews) guidelines [44] were followed to ensure appropriate reporting.

### Search Strategy and Eligibility Criteria

A search strategy was developed and used a search string consisting of words related to dementia; technology designed to support health, care, or well-being; and PPI. Knowing that the terminology used varies considerably, definitions were kept broad, in particular of “patient and public involvement,” adapting and building on existing search strings from previous reviews [11,19,25,41,42]. Our definition of technology was similarly broad. Assistive technology may be described as “products or systems that support and assist individuals with disabilities, restrict mobility or other impairments to perform functions that might otherwise be difficult or impossible” [46]. We included any type of assistive technology as well as, more broadly, any technology that could be deemed to be a part of technology-enabled care (such as telehealth systems, telecare, telemedicine, and self-care apps) [47]. Inclusion criteria were developed (Textbox 2). PPI activities do not usually require ethics approval [20], yet we did not exclude those who sought ethics approval so as to ensure that we captured a range of approaches.

**Inclusion and exclusion criteria.**

**Inclusion criteria**

- Research about dementia (any type) or mild cognitive impairment
- Research focused on technology designed to support the health, care, or well-being of people with dementia or their carers
- Research describing ways in which patients or other stakeholders were actively involved in the research process itself (not only as research participants)
- Full text available in English
- Any publication date up to the end of 2022

**Exclusion criteria**

- Dementia only mentioned incidentally (eg, primary focus was Parkinson disease)
- Technology in which target beneficiaries are not people with dementia, family or carers (eg, web-based education programs for health care workers)
- Studies in which the patients or stakeholders are positioned as research participants only (eg, participants in a qualitative study) and are not actively involved in conducting the research
- Reviews
- Opinion pieces
- Conference abstracts

### Data Sources and Charting Process

The search was conducted in 4 databases: MEDLINE, PsycINFO, Embase (using Ovid), and CINAHL (using EBSCO). All papers published until the end of 2022 were included. Abstracts had to be available in English, and opinion pieces and reviews were excluded (refer to Multimedia Appendix 1 for the full MEDLINE search string). The search was last conducted in January 2023. References were exported to EndNote (Clarivate Analytics) and then to Covidence (Veritas Health Information) [48] for screening. After the removal of duplicates, 2 reviewers (PK and HL) screened the titles and abstracts against the eligibility criteria. The full texts were then further screened for eligibility. The 2 reviewers then independently charted the data from the included studies using a predesigned extraction table. For the first 10 papers, detailed discussions were held to clarify interpretations of PPI. Subsequent discussions were held to reach a consensus where required. A third author (CMA) was available if a consensus was not reached. As the purpose of this review was to provide an overview of existing evidence regardless of quality, no formal appraisal of methodological quality was conducted, in line with guidance [45]. Facilitators of and barriers to effective PPI were grouped and analyzed by the first author to draw out themes, which were then refined in discussion with the other authors. The impact of PPI, where described, was summarized and categorized into impact on the study, impact on the patient and public partners, and impact on the research team.

**Figure 1:**
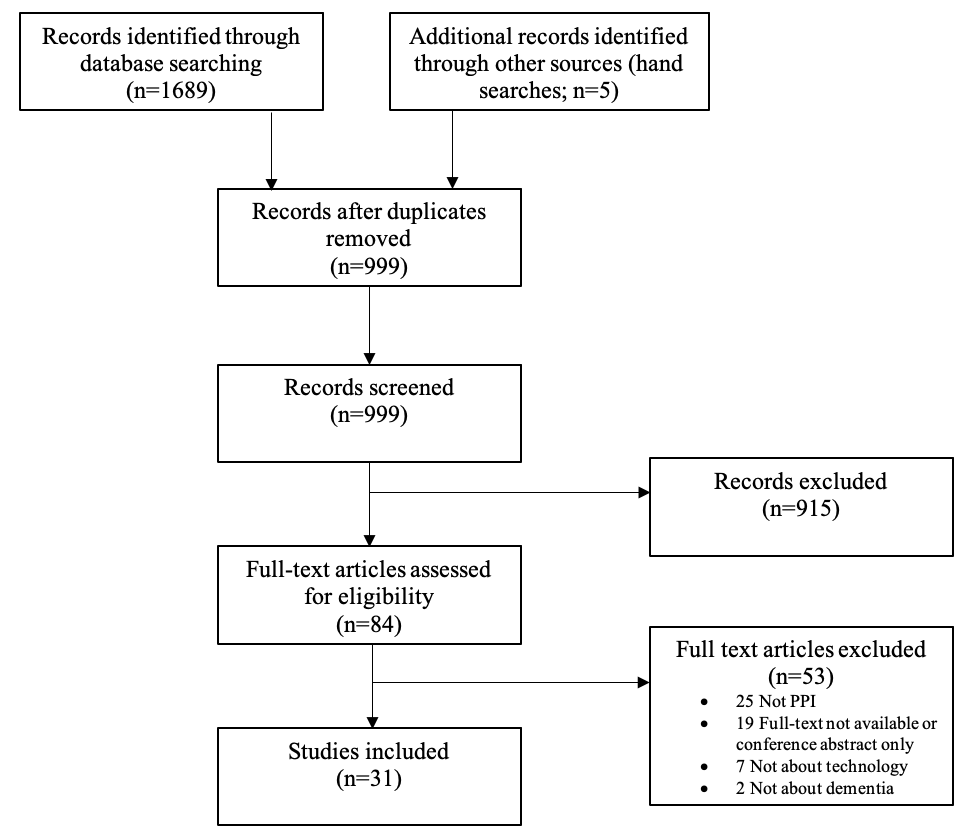
Record identification and screening process. PPI: patient and public involvement.

Initial database searching identified 1689 records, with an additional 5 found through hand searches following references from papers identified in the initial search. After removal of 695 (41%) duplicates, the remaining 999 abstracts were screened. Most of these (915/999, 91.6%) did not meet the eligibility criteria (were not about dementia, involvement in research, or technology). Determining whether papers described active involvement in the research process or merely involvement as participants was frequently unclear from the abstracts alone, and the authors erred on the side of inclusion here, in line with guidance. When analyzing full texts (84/999, 8.4%), not meeting the “involvement in research” criteria was the most common reason for exclusion (25/53, 47% of the papers excluded at this stage). A total of 31 papers were included in the scoping review. Figure 1 shows the flow of information for this process.

## Results

### Study Characteristics

Of the 31 papers included in the review (Table 1), most (n=18, 58%) were published between 2020 and 2022. Only 13% (4/31) were published before 2010. Most papers were authored by groups from multiple disciplines, for example, authors from design engineering backgrounds and health sciences and health care professionals. A total of 13% (4/31) of the studies included patient or public partners as coauthors [17,49-51]. In total, 21 of the studies originated in Europe, including 10 (48%) from the United Kingdom. Others were from Canada (7/31, 23%) and Australia (3/31, 10%), and 1 of the studies (1/31, 3%) included discussions of collaborations with groups in Ireland, Hong Kong, Brazil, and India [52].

Table 1 also outlines the stakeholders involved, the stage or stages of the research they were involved in, and the methods used for this involvement. When determining which stage of research stakeholders were involved in, the authors mapped involvement onto NIHR stages of research in which PPI might typically take place (eg, “design of the research”). Where Table 1 states “did not fit model,” this was because participants were positioned as both coresearchers and research participants. This is explored further in the following sections (Table 2).

A range of technologies were described with varied purposes (Textbox 3), apart from in Liddle et al [17], which did not focus on any one type.

**Table 1:** Study characteristics describing the technology, stakeholder involvement, stage of the research process in which the involvement took place, role of the stakeholder, and methods used to involve patients and the public.

Study, year	Origin	Technology type and purpose	Stakeholders involved	NIHR^a^ stage of research in which PPI^b^ took place	Role of stakeholders involved	PPI methods
Davies et al [53], 2019	United Kingdom	Website to provide information and peer and professional support for caregivers of people with dementia toward end of life	Research development group including 6 HCPs^c^, 2 members of a dementia charity organization, and 1 carer	Design of the research, undertaking and management of the research, and analysis of data	Refining the aims of the wider project and steering the project throughout	Steering group, group meetings, and 1:1 meetings
Kort and van Hoof [54], 2014	The Netherlands	Website with information about home modifications for people with dementia and their family caregivers	3 dyads (people with dementia+carer) and, separately, a group of 20 (carers or residential home staff)	Did not fit model	Contributing to the iterative design process	Observations, consultation rounds, and questionnaire
Schikhof et al [55], 2010	The Netherlands	Monitoring system in residential home for people with dementia to detect anomalies (eg, panicking and falls)	8 nursing home staff members and 13 family representatives (as proxies for people with dementia)	Did not fit model	Contributing to the iterative design process	Interviews, workshops for prototype testing, informal group meetings, and focus groups
Muñoz et al [56], 2022	Canada	Virtual reality game to support engagement in exercise for people with dementia	7 people with dementia or MCI^d^, 5 older adults without dementia, industry partners, and HCPs	Design of the research and undertaking and management of the research; elements did not fit model	Contributing to the iterative design process; HCPs and industry representatives also had a role in designing and managing the study	Prototype testing and interviews (people with dementia or MCI and older adults), research group meetings (HCPs), and focus group (older adults without dementia, members of the research team, and industry representatives)
Eisapour et al [57], 2020, also with reference to Eisapour et al [58], 2018, and Eisapour [59], 2018	Canada	Virtual reality game to support engagement in exercise for people with dementia	HCPs and 3 people with dementia	One PPI representative involved in the main research team, presumed to be throughout; other elements did not fit model	Contributing to the iterative design process and involved in the main research team	Focus groups, observations, informal discussions in care home, and prototype testing; one member of the original focus group subsequently joined the research team
Hanson et al [60], 2007	Sweden	Home computer-based education and support program providing information, support tools, and exercises for people with dementia and their carers	7 people with dementia	Did not fit model	Contributing to the iterative design process	Group meetings to develop support program content and computer skills tuition for group members
Orpwood et al [61], 2004	Finland and others part of the ENABLE project	Various assistive technology devices: picture gramophone, calendar, tap monitor, lost object locator, gas cooker monitor, and night-light	Family carers (as proxies for people with dementia), paid carers, and older adults without dementia	Did not fit model	Contributing to the iterative design process	Informal group meetings and focus groups
Kort et al [62], 2019	The Netherlands	Various projects described: a smart pill box and real-time location systems (also a website as documented separately [51])	People with dementia and carers (past or current)	Did not fit model	Contributing to the iterative design process	Varied between projects: observations, consultations, storytelling, focus groups, and “thinking aloud” sessions
Hendriks et al [63], 2017, also with reference to Hendriks et al [64], 2014	Belgium	System for monitoring the mealtimes of people with dementia in a care home using sensors and data input by carers into the app	Industry representatives, academics and HCPs, professional carers, people with dementia, and informal carers	Did not fit model	Contributing to the iterative design process	Meetings, feedback sessions with HCPs or professional carers, integration of research team into daily life at care home, and group “mapping” sessions with people with dementia and carers
Orpwood et al [65], 2007	United Kingdom	Various technology projects: music player, video streaming of outside world scenes into the homes of people with dementia, conversation prompter for people with dementia, and “sequence support” tool for prompting ADLs^e^	Varied across projects: people with dementia; carers; and other “user representatives,” including academics from the social sciences, engineering, and dementia organizations	Did not fit model	Contributing to the iterative design process	Varied across projects: user survey, co-design workshops, observations, 1:1 user testing, and interviews
Savitch et al [66], 2012	United Kingdom	Website providing information about assistive technology for people with dementia	People with dementia and, separately, a steering group that also included 1 person with dementia	Steering group: detail not given; elements did not fit model	Contributing to the iterative design process (people with dementia); steering group also provided input throughout	Interviews, focus groups, co-design workshops, and involvement in steering group meetings
Perkins et al [52], 2022	United Kingdom, Ireland, Hong Kong, India, and Brazil	Web-based delivery of CST^f^	4 people with dementia, 4 family carers, 4 service managers, and 8 CST group facilitators from the United Kingdom and Hong Kong; additional stakeholders in India	Design of the research and undertaking and management of the research	Designing and developing a protocol (people with dementia, carers, service managers, and CST facilitators) and field-testing of the protocol and subsequently giving feedback following testing (CST facilitators)	Focus groups (web-based); CST facilitators then tested the protocol with people with dementia in 5 countries; interviews with CST facilitators following field-testing
Hwang et al [67], 2015	Canada	Animated videos for prompting people with dementia with ADLs	6 family carers	Did not fit model	Contributing to the iterative design process	Co-design workshops first to develop the concept and then refine the designs; 2 home visits for paper prototype evaluation
Oksnebjerg et al [68], 2019	Denmark	An app to support self-management for people with dementia, including a calendar and diary	4 people with dementia, 4 family carers, and 4 paid carers	Did not fit model	Contributing to the iterative design process	Co-design workshops
Hung et al [50], 2021	Canada	PARO, a commercially available robot seal that uses AI^g^ to support the social and emotional needs of the people with dementia interacting with it	5 “patient and family partners”	Undertaking and managing the research, analysis of data, and dissemination of research findings	Supporting data collection, thematic analysis of data, and authoring of the paper	Supporting data collection with some patients or particular settings, analysis (4 × 1-h thematic analysis group sessions), and coauthoring the paper
Rathnayake et al [37], 2021	Australia	A mobile health app that provides information about dementia, care strategies, and tips for managing ADLs	Carers, HCPs, and IT experts	Did not fit model	Contributing to the iterative design process	Web-based survey, interviews, and co-design workshops
Kowe et al [69], 2021, also with reference to Kowe et al [70], 2022	Germany	Sensor-based activity management system	6 family carers	Analysis of data	Supporting thematic analysis of interview data	30-min group analysis session or workshop
Daly Lynn et al [51], 2021, also with reference to Daly Lynn et al [71], 2019, and Daly Lynn et al [72], 2022	United Kingdom	Smart home system for people with dementia in supported living	Peer researchers: 7 older adults without dementia; steering group: including 2 people with dementia, 1 family carer, and 2 dementia organization employees	Undertaking and management of the research, analysis of data, and dissemination; steering group: detail not given	Peer researchers: conducting interviews with research participants and supporting data analysis; steering group: ensuring that the voice of older people was central to the project and coauthoring the paper	2-d training for peer researchers, conducting interviews jointly with a member of the research team, group thematic analysis session, and evaluation forms
Banbury et al [73], 2021	Australia	Virtual support program to provide information and peer support for carers of people with dementia	6 family carers	Did not fit model	Contributing to the iterative design process	Co-design workshops and group meetings (all virtual) following the Double Diamond approach
Fox et al [74], 2022	United Kingdom	A mobile health app that delivers memory tests throughout the day to monitor cognition changes	7 people with dementia, 7 family carers, and a PPI officer	Did not fit model	PPI officer as “proxy patient representative” in the research team; people with dementia and carers contributed to the iterative design process	Co-design workshops; PPI officer part of the main research group
Rai et al [75], 2020	United Kingdom	Virtual delivery of CST	People with dementia and family carers	Did not fit model	Contributing to the iterative design process	Consultation meetings and co-design workshops
Owens et al [76], 2020	Consortium spanning organizations in Europe and the United States	Remote monitoring and measurement technologies for people with dementia	People with dementia and family carers	Design of the research	Patient advisory board—supporting research planning and design and feedback on relevance and priorities	Provision of feedback on researchers’ literature review, group discussions, workshops, and other meetings of the patient advisory board
Stavropoulos et al [77], 2021	Greece, with involvement from multiple countries across Europe	A range of commercially available wearable devices	11 people with dementia and 10 carers from 11 countries across Europe	Design of the research	Reviewing devices and providing information to guide the design of future research, aiming to make it more relevant and accessible and improve participant experience	One-off 2.5-h session including presentations, roundtable discussions, hands-on experimentation, and voting
Liddle et al [17], 2022	Australia	No specific technology discussed—study explored factors related to engagement with technology for people with dementia and carers	15 people with dementia and carers (living experience expert reference group); 4 of them (2 people with dementia and 2 carers) were also members of the core research team and were listed as coauthors	Design of the research, undertaking and management of the research, analysis of data, and dissemination of research findings	Setting research priorities, supporting the design of interviews and developing the topic guide, thematic analysis of data, reflexivity sessions with the research team, and writing up of the study	Group sessions, discussions and meetings with reference group, group analysis sessions, and discussions of reflexivity
Hassan et al [78], 2017	United Kingdom	A range of commercially available wearable devices	>30 in total: people with dementia or MCI, carers, people with early-onset dementia (aged <65 y), and older adults without dementia	Design of the research	Contributing to research design (producing guidelines to optimize design and improve acceptability) and informing procurement decisions	Exploratory workshops in groups according to diagnosis (people with dementia+carers, people with early-onset dementia, and older adults without dementia), individual 1:1 meetings (people with MCI), and opportunities for stakeholders to try the devices at home
Jacklin et al [79], 2020	Canada	A wearable (wristband) for capturing movement-related behaviors (eg, falls, wandering, and agitation)	Indigenous community representatives, carers, community elders, and health and social care workers	Did not fit model	Informing the design of the research to ensure that culturally appropriate and inclusive methods are used	Community-based participatory research approach, focus groups (with carers), and preparation meetings with the Community Advisory Council to plan the research and ensure that appropriate methods are used
Ghafurian et al [80], 2022	Canada	App to support professional carers in communicating with people with dementia in nursing homes	17 professional carers and 1 nurse	Did not fit model	Contributing to the iterative design process	Survey, focus groups, and co-design workshops
Berge et al [81], 2022	Norway	Tablet-based music app primarily used as a relational tool to support positive interactions between people with dementia and carers	People with dementia or other psychiatric conditions, carers, and HCPs	Did not fit model	Contributing to the iterative design process	Observations, interviews, and 2 focus groups; user testing of a prototype with 4 older adults with dementia or other psychiatric conditions
Shadarevian et al [49], 2020	Canada	Tablet for sharing family videos in hospital with people with dementia to support care provision, reduce social isolation, and reduce aggression	People with dementia, family carers, HCPs, and students	Undertaking and management of the research, analysis of data, and dissemination of research findings	Positioned as part of the research team throughout, guiding the research process; thematic analysis of interview data; creating a toolkit to support wider dissemination and adoption of the intervention; and coauthoring the paper	Monthly research project meetings, making videos, interviews, group thematic analysis session, manuscript reviewing and editing
Tiersen et al [82], 2021	United Kingdom	Smart home system for people with dementia in their own homes	People with dementia, carers, and HCPs	Did not fit model	Contributing to the iterative design process	Various across 9 substudies: semistructured interviews, focus groups, co-design workshops, surveys, home visits, online group meetings, and observations
van der Roest et al [83], 2008	The Netherlands	Website with customized information for people with dementia and their carers about health care and welfare services	People with dementia, carers, and HCPs	Did not fit model	Contributing to the iterative design process	Workshop exploring user needs (people with dementia, carers, and HCPs), further co-design workshops (HCPs), prototype testing, and questionnaire (family carers)

^a^NIHR: National Institute for Health and Care Research.

^b^PPI: patient and public involvement.

^c^HCP: health care professional.

^d^MCI: mild cognitive impairment.

^e^ADL: activity of daily living.

^f^CST: cognitive stimulation therapy.

^g^AI: artificial intelligence.

**Table 2:** Patient and public involvement (PPI) in the different stages of research as recommended by the National Institute for Health and Care Research [21] (n=31).

Stage of research	Studies with PPI at this stage, n (%)	Example
Design of the research and development of the grant application	7 (23)	PPI representatives (here a “Patient Advisory Board”) supported research planning and design, giving feedback on relevance and priorities [76].
Undertaking and management of the research	7 (23)	PPI representatives, here termed “peer researchers,” collected data for the study interviewing people with dementia [51].
Analysis of data	6 (19)	PPI representatives were part of interview data analysis and discussions of reflexivity [17].
Dissemination of research findings	4 (13)	PPI representatives coauthored the paper [49].
Did not fit model	21 (68)	PPI representatives were positioned both as co-designers along with the research team and as research participants testing the prototype [81].

**Types of technologies and their purposes.**

**Sensor monitoring systems (including smart home monitoring systems) [51,55,63,69,76,82]**

- Safety alerts
- Monitoring (of health, activity, behavior, and cognition)

**Wearables [77-79]**

- Safety alerts
- Monitoring (of health, activity, behavior, and cognition)

**Apps [37,60,68,74,80,81]**

- Monitoring (of health, activity, behavior, and cognition)
- Self-management
- Exercises
- Information provision or education
- Supporting social interaction
- Social and emotional well-being

**Miscellaneous assistive technology devices (not wearables and not app based, eg, gas cooker monitor or smart pill box) [50,61,62,65,67]**

- Safety alerts
- Self-management
- Leisure access
- Supporting social interaction
- Social and emotional well-being

**Websites [53,54,66,83]**

- Information provision and education
- Accessing peer support
- Accessing professional support

**Videoconferencing platforms [73,75]**

- Therapy delivery
- Information provision and education
- Accessing peer support

**Virtual reality games [56,57]**

- Exercises

### Who Was Involved?

Studies involving only 1 stakeholder group in their PPI activities were in the minority (5/31, 16%), and many (17/31, 55%) involved ≥3 different stakeholder groups, with the range of these shown in Multimedia Appendix 2. Family carers were the most frequently involved group (27/31, 87% of the studies), followed by people living with dementia (23/31, 74%). Most papers gave little detail about recruitment methods or the background of their PPI representatives. Where papers mentioned attempts to recruit diverse viewpoints, this generally referred to involving different stakeholder perspectives (eg, patients as well as carers and health care professionals), and where inclusivity was dwelled on, this usually referred to the involvement of people living with dementia. Some papers mentioned the linguistic mix or geographical spread of those involved, in particular [52,76,77]. Discussions of racial or ethnic diversity within PPI groups were almost entirely absent. There was one exception [79] in which the inclusion of First Nations representatives was central to the study.

### When Were They Involved?

The NIHR recommends PPI throughout the research cycle, highlighting in particular 4 key areas where PPI can take place [21]. The studies in this review were mapped to these stages, as shown in Table 2. In total, 29% (9/31) of the studies had involvement from patient and public partners at multiple stages throughout the research cycle [17,49-53,56,57,66], although sometimes a lack of detail on methods meant that this multistage involvement was presumed (eg, description of a steering group providing oversight “at key milestones” [66] without further description).

As shown in Table 2, a total of 68% (21/31) of the sources did not fit into this NIHR description. These were papers describing a co-design or participatory design process in which the stakeholders involved were both the “co-researchers” or “co-designers” and yet were also positioned as research participants. Typically, these studies involved stakeholders in the iterative design process of a technology prototype. Participants collaborated with the research team on the design process while also being positioned as study participants, for example, being observed testing prototypes or providing feedback as part of interviews. Their involvement could not clearly be classified as designing the research (the study protocol having been designed before their input) or quite as “undertaking/managing the research” as they were the targets of data collection, not involved in the process of collecting them themselves. However, as the authors positioned these stakeholders as collaborators or co-designers along with the research team, these studies were not excluded in the same way that others were when they were more clearly set up as qualitative studies (eg, a focus group to collect end users’ views on technology where ethics approval had been sought for this research process).

### How Were They Involved?

Approaches to PPI varied (Table 1). Every paper mentioned at least one form of group activity for their involvement work. Varying terms were used for this—co-design workshops and focus groups were the most frequently mentioned, along with group meetings, group discussions, prototype testing sessions, consultation rounds, group consultations, informal meetings, group feedback sessions, and workshops. Most papers (26/31, 84%) described more than 1 type of activity. In addition to group activities, many conducted interviews [37,49,51,52,55,56,65,66,81,82], observations [54,57,62,65,81,82], or surveys or questionnaires [37,54,65,80,82,83]. A total of 19% (6/31) of the studies set up steering groups that were regularly involved in the research process, described variously as a research development group [53], living experience expert reference group [17], steering group [51,66,71], and patient advisory board [76,77], although details were minimal or absent about what this entailed. A total of 19% (6/31) of the papers [17,49-51,57,74] described some form of integration of patient and public partners (or, in 1 case, a PPI officer as proxy for the PPI group itself [74]) into the main research team, although, again, details were often very minimal about what this entailed. In total, 13% (4/31) of the papers were coauthored by patient and public partners [17,49-51].

### Barriers to and Facilitators of Effective PPI

A total of 74% (23/31) of the papers included at least some reporting of either facilitators or barriers faced when conducting PPI. In many cases, this reporting was minimal, for example, listing one challenge the team faced. Only 26% (8/31) of the papers had what we considered to be a more thorough discussion of barriers or facilitators [51,56,60,63,64,69,70,73,74,78] (the papers by Hendriks et al [63,64] refer to the same study, as do those by Kowe et al [69,70]). Barriers and facilitators were grouped into themes (Textboxes 4 and 5). Facilitators often focused on ways to achieve richer, more meaningful involvement, for example, working with multiple stakeholder groups and creating a trusting, supportive group dynamic. The barriers identified principally regarded issues with working with dementia as a condition as well as practical issues such as time and budget.

**Facilitating effective patient and public involvement (PPI).**

**A person-centered approach: choices and adaptability in involvement**

1. Prioritizing the well-being and positive experience of those involved [60,77]
2. Offering choices and being led by those involved (how to take part, methods, environment, and level of involvement) [17,69,82]
3. Use of extra time and flexibility for people with dementia, including modification of activities to make them more accessible, acknowledging that there will be no *one-size-fits-all* [60,77]

**Building the group: rapport, trust, and equality**

1. Spending time developing group relationships, finding commonalities, and building connections within the team [51,73,77]
2. Building time for chatting and eating together. Informality helps build rapport and flatten hierarchies [60,63,65]
3. Use of a nonresearcher as facilitator [69]
4. Being face-to-face rather than web-based [77]
5. Use of small groups [60]

**Multiple viewpoints**

1. Including views from multiple stakeholder groups as a way of improving the quality of involvement work and the richness of the data gathered [78,82]
2. Planning a range of methods to recruit and work with different groups (carers, people with dementia, health care professionals, and others) seen as important [82]
3. Considering ways of involving seldom heard groups—from practical adaptations (researchers traveling and not asking patient and public partners to do so) [80] to cultural considerations [79]
4. Group members from different backgrounds learning from each other [65]
5. Support to access different groups was beneficial (eg, working with community organizations or having managerial support to enable staff to take time away from their main role [56])

**The right environment**

1. Considering accessibility and proximity to local amenities and transport [60,78]
2. Considering who owns the environment—researchers going to those involved (eg, integrating into nursing home environment) may help create a greater sense of equality, flatten hierarchies, and support researchers’ understanding of the group they are working with [56,63,80]
3. Being face-to-face enabled hands-on workshops, improved group dynamics, and reduced technology barriers [77,78]
4. Web-based environments enable geographically diverse groups to come together and may keep discussions more focused [73]

**Support and training**

1. Having facilitators or members of the research team who are skilled and experienced working with people with dementia [60,78]
2. Providing training for patient and public partners (eg, data collection, thematic analysis, and computer skills) [51,60]
3. Supporting patient and public partners with adequate time to reflect and debrief with members of the academic research team [51]
4. Using paper prototypes to overcome technology barriers [66]
5. Providing adequate support for people with dementia (family carers [60] or modified activities [60,77,78])

**Pragmatism and compromise**

1. Proxy involvement (of family, PPI officers, and nursing home staff) used in place of people with dementia (or people with moderate to severe dementia) in cases in which their involvement was not seen as feasible [55,61,74]
2. One-to-one sessions found to be easier to organize than group sessions [53]
3. Virtual meetings may be easier to organize than face-to-face meetings [73]

**Barriers to effective patient and public involvement (PPI).**

**The nature of dementia**

1. Cognitive impairments seen as too great a barrier to attempt PPI with people with dementia [55,61]
2. Input from people with dementia described as very minimal [62]
3. Attempts at adaptations unsuccessful [63,64]
4. Variations in presentation making it difficult to plan a particular approach or manage a group [63,64]
5. Carers and people with dementia both overestimating the abilities of the latter [63,64]
6. Unreliable historians— for example the challenge of interpreting someone’s account of their ability to participate in activities of daily living while they also recount recent interactions with long-dead relatives [63,64]
7. The emotional load faced by researchers working with this group, including challenges such as being asked for support or advice out of their scope [63,64]

**Inequality of relationships within the group**

1. Some authors highlighted issues with patient and public partners feeling undervalued or not equal within the team; this applied to those without dementia [51,63], though dementia was also seen as an additional barrier to a sense of equality [63,64]
2. Lack of payment for PPI also contributed to this, as well as the limited scope or lack of defined roles and responsibilities for patient and public partners [63,69]

**Time pressures**

1. Researchers’ time pressures—co-design or other involvement activities as time-consuming processes that can be difficult to manage alongside the time pressures of a research study [75,81,83]
2. Family carers’ time pressures—busy schedules and stressful lives [37,69]
3. Staff time pressures—nursing home staff and health care professionals’ strict shift patterns and limited flexibility for time away from work [80], in some cases exacerbated by the COVID-19 pandemic [52]
4. Limited time resulting in reduced or inadequate training for patient and public partners [51,69]
5. Rapport building in the group suffering as a result of lack of time [51,56]

**Recruitment and diversity**

1. Small numbers of people involved resulting in reduced diversity of opinions and a poorer representation of stakeholders [56,57,75]
2. Challenges with generating interest in the study or reaching particular groups [74,78] and COVID-19 causing staffing pressures [52] and a lack of face-to-face options for people with dementia [82]

**Processes and communication**

1. Communicating complex content (the ethics of smart homes, technology use, and design processes) was particularly challenging for people with dementia [62,68]
2. Use of jargon terminology by researchers was a barrier for all patient and public partners (not only those with dementia) [63,69]
3. Methods of communication—use of phone for people with dementia was limiting [82], and sending too many emails was unpopular [56]
4. Processes for PPI members experienced as boring or repetitive (eg, completing multiple assessments), especially when combined with a lack of communication about the purpose or the results of their input [56]
5. Lack of involvement and communication early in the study resulting in stakeholders having less of a connection or understanding of the project or feeling that their contributions were less valued [51,63]

### Impact of PPI

Although most papers (28/31, 90%) implied or briefly commented that stakeholder involvement had some impact on their study (usually on the iterative design process), this was sometimes without any description of what the impact was. Where any details were given, as was the case in 52% (16/31) of the papers [51,54-57,60,61,63,69,73-79], the results are summarized in Textbox 6.

**Impact of patient and public involvement (PPI) activities.**

**Impact on the research**

- PPI activities helped set groups’ research agendas, with clearly defined stakeholder priorities for research [76,77]. PPI data were identified as something that can be shared with and used by the wider research community when planning research [77].
- Involvement in research design resulted in a set of recommendations that the authors hope will improve the acceptability for research participants [78] and in specific cultural adaptations and approaches [79].
- Involvement in data collection was reported as adding richness to the data on account of the rapport and connections that peer researchers built with the people with dementia they were interviewing [51].
- Many papers (21/31, 68%) commented that the methods used (eg, co-design and participatory design) had an end result that was in some way grounded in the views or priorities of users but often with minimal detail. In total, 13% (4/31) of the papers [55,57,74,75] gave detail about the extent to which user groups drove the development or design of technology, reflecting on the value of their input.
- Coresearcher involvement in a thematic analysis workshop made for a more robust analysis, with differing perspectives between the research team and coresearchers showing the need for more PPI at the analysis stage in the future [69]. The limited impact that PPI activities had at the analysis stage was also reflected on, citing inadequate time and training for coresearchers resulting in brief and surface-level group analysis sessions [51,69].

**Impact on patient and public partners**

- Feedback on positive experiences of patient and public partners was provided in general terms [74,78]. Positive relationships between team members were developed, with feelings of mutual respect as well as the value of finding connections being reported [51,56,73].
- Some reported empowerment and satisfaction with the project and their role in it [54,60,61].
- Patient and public partners developed new skills [51].
- Negative experiences were reported on, including finding tasks boring or repetitive or processes complex [56]. Some papers also reported that patient and public partners felt underinvolved [51] or not treated as equal partners [63].

**Impact on the academic research team**

- Researchers gained a deeper understanding of the needs and priorities of the group they were seeking to conduct research with and for [79].
- Researchers developed a sense of connection with and respect for other disciplines or stakeholders they had not previously worked with [60,65].
- One paper reflected on the emotional burden associated with close working with people with dementia and the need for support for researchers as well as the people with dementia themselves [64].
- The initial challenges of stepping back when sharing responsibility with peer researchers was reported on, which became easier with experience [51].

In general, no formal methods were used for evaluating the impact of PPI activities. Where papers reported on impact, it was usually limited to the authors’ reflections, including when reporting on the impact on patient and public partners. In the case of 13% (4/31) of the papers [51,56,63,73], the authors reported seeking direct feedback from those who had been involved, for example, in the form of interviews; evaluation forms; or, in 3% (1/31) of the studies, a much more extensive retrospective analysis using formalized methods [63].

## Discussion

### Principal Findings

In this scoping review, we set out to explore the concept of PPI in technology-related dementia research. The papers reviewed in this study revealed that dementia researchers are embracing PPI, with varied and sometimes ambitious methods, values centered on inclusivity and coproduction, and involvement of a range of stakeholder groups. We found that approaches often blurred boundaries between those involved as “researchers” and those involved as “participants” so that most studies’ (21/31, 68%) PPI activities did not fit into a strict definition of PPI, for example, as set out by the NIHR [32]. Although the involvement activities being undertaken demonstrate this to be a rapidly expanding and developing field, the brevity in the reporting of such activities (often without comments on the impact of PPI) perhaps highlights the need for clearer reporting guidelines. Where mentioned, the impact of PPI was generally reported as being positive on research quality, patient and public experience, and the learning experiences provided to researchers. We comment further on our objectives in the following sections.

### Objective 1: To Describe the Approaches to PPI Used to Date in Technology-Related Dementia Research (Exploring Who Is Involved, When, and How)

We found that there was a narrative across many of the included papers about the value of involvement and coproduction methods, with many authors describing their aspiration for high levels of involvement with a sense of partnership and equality with stakeholders. A few consciously excluded people with dementia from this aspiration, citing cognitive impairments as making it either practically or ethically too challenging to involve this group. These views were chiefly expressed in older papers (before 2010). More recent papers were broadly inclusive, with some describing their efforts to involve people with dementia along with other stakeholder groups such as carers, health care professionals, and older adults without dementia. Sometimes, these groups were involved in similar ways, and sometimes, there were 2 very separate approaches, for example, a set of workshops with people with dementia and carers and more extensive involvement of health care professionals or others without dementia in the research process (eg, playing a role in designing the protocol or as members of a steering group). The fact that a significant majority (26/31, 84%) involved more than one stakeholder group, with many involving ≥3 groups (17/31, 55%), reflects the value placed on hearing from multiple viewpoints.

Despite this widespread acknowledgment of the value of collaborative or coproduction methods, it was not always clear from the papers to what extent their methods reflected these values. Some used methods that perhaps lend themselves better to a consultative approach (such as one-off focus groups or surveys) rather than a collaboration or coproduction approach [20]. Consultative methods have some value in enabling researchers to find out more about people’s views and experiences. They are also relatively easy to organize (often one-off events as opposed to longer-term involvement), meaning that they are a practical way of hearing from a wide range of stakeholders [20]. However, these methods mean that involvement will always be more limited in scope—there is no 2-way discussion, and there is the risk of disengagement from stakeholders who feel that their views are not being listened to [20], as indeed was reported by Hendriks et al [63]. Where stated methods or approaches may have enabled more of a partnership approach to take place (eg, the use of a series of co-design workshops or involvement of a steering group), the brevity of the write-up often prevented us from understanding whether or how this happened.

The most common methods of involvement were through some form of group activity such as focus groups, workshops, prototype testing sessions, or roundtable discussions. Every paper mentioned some form of group activity such as these, with or without other methods. Involvement often occurred at just one stage of research—most commonly, this was contributing to the design of a prototype but with no involvement either before or after this (eg, in protocol design, recruitment, analysis, or dissemination), although there were exceptions to this, with studies involving patients or the public throughout multiple stages of the research.

Most studies (26/31, 84%) used multiple methods of involvement. There will be practical reasons for using different methods, some suiting particular groups or settings more than others, especially when we consider involving people with dementia and the flexibility of approach that this requires. Tiersen et al [82] described many different methods in their paper, reflecting that this “resulted in triangulation of investigators, methods and data sources to develop a more comprehensive understanding of the phenomena being studied.” The use of multiple methods also allowed for more flexibility of involvement, with some able to take on a more active or sustained role than others as able or desired. This was cited as an aspiration or suggestion for future PPI by groups who did not have such flexible approaches, such as Kowe et al [69]. The paper by Liddle et al [17] described the flexibility of roles and high levels of involvement. Their “Living Experience Expert Reference Group” involved 15 people with dementia and carers, of whom 4 (2 people with dementia and 2 carers) were also integrated into the main investigator team. Roles included helping set research priorities, developing the interview topic guide, analysis of data, reflexivity sessions, and the write-up of the study. Shadarevian et al [49] and Hung et al [50] also described the integration of stakeholders into the main research group, mentioning roles in undertaking and managing the research along with analysis and dissemination, although there was little detail about the methods used for this involvement. PPI in data collection was rare. It was described in most depth by Daly Lynn et al [51], who worked with “peer researchers” (older adults without dementia) to interview research participants with dementia about their experiences with smart home living, with an insightful write-up detailing both the positive experiences and the challenges they faced.

Finally, although the notion of diverse viewpoints being included in PPI was often celebrated, this almost always referred to the inclusion of a variety of stakeholder groups (eg, patients, carers, and health care professionals). In general, there was very little explicit discussion of the demographics of PPI representatives. To reduce health-related inequalities and bias, researchers should consider not only how to involve people with dementia but also the demographics of this heterogeneous population, explicitly seeking ways to involve underrepresented groups.

### Objective 2: To Describe the Reported Barriers to and Facilitators of Effective PPI in This Area

The general lack of evaluation or reflections on barriers to and facilitators of involvement means that the themes described in this paper result from a minority of studies, with most derived from just 26% (8/31) of the studies [51,56,60,63,69,73,74,78]. Themes drawn out broadly matched those frequently documented in the literature [11,25,41,42], in particular barriers such as time and budget, recruitment issues, and the specific challenges of adapting activities to be suitable for people with dementia.

Facilitators tended to focus on the manner in which activities were carried out (eg, how informality helped flatten hierarchies) rather than on specific methods or approaches, such as focus groups or interviews. The themes here mirror the key principles of coproduction outlined by the NIHR [21,32]. The NIHR emphasizes that coproduction does not require a specific method but a more nuanced focus on interpersonal skills, relationship building, and power sharing (Textbox 1) [32]. However, although many papers stated the need for approaches using these principles or claimed to have worked with such values in mind, details about what was done were often limited. We would welcome further and more detailed reporting on these activities so as to build the knowledge base among research teams and enable more high-quality PPI to be conducted in the future with this population. As also emphasized by Hendriks et al [64], there is a need for more than anecdotal evidence in the literature about how to involve people with dementia—the lack of guidelines or a strong evidence base makes progression challenging.

The barriers identified reflect the challenging nature of PPI in technology-related dementia research. Time and resources were frequently cited as limiting factors. Some studies avoided the challenge of making PPI activities accessible to people with dementia by not involving them at all. Hendriks et al [64] detailed the challenges of involving people with dementia in a particularly frank manner. They reported on the difficulty of sufficiently modifying activities to make them accessible, difficulties with variability in dementia presentations, and overestimation of abilities by people with dementia and their family carers. They went as far as to say that “the differences between the designer and the person to design for are too big to speak about equality in participation” [64]. A few papers highlighted the complex topics under discussion (eg, smart home technology, ethics, and design processes) and the difficulty of translating these issues into something someone with dementia could understand and contribute to [62,63,68]. Kort et al [62] went as far as to say that the complexity of content meant that input from people with dementia was very basic, commenting that “the actual participation in the project was deemed more important than the actual contribution.” Although pessimistic sounding in tone at times, these honest admissions of the challenges faced and the inability of researchers to overcome them to a meaningful degree help the research community understand the current landscape of PPI in technology-related dementia research. They reinforce the need for significant investment to be made for the involvement of people with dementia to be successful. This is not merely in the creation of accessible resources or the provision of the right environment. Rather, it may be that significant cultural shifts need to take place for researchers to be able to plan and conduct effective coproduction based on the key principles of power sharing, inclusivity, respect, reciprocity, and relationship building [32]. It seems likely that researchers would benefit from significant training and support in understanding power dynamics and coproduction as well as support to learn more about how to work with people with cognitive impairments.

### Objective 3: To Examine and Report on the Impact of PPI in This Area

Papers with more than a brief comment on the impact of involvement were scarce in this review, mirroring the findings by Suijkerbuijk et al [41] as well as the findings of those studies considering PPI in dementia research more broadly [25,26,42,84]. A few papers, notably those by Daly Lynn et al [51], Muñoz et al [56], Banbury et al [73], and Hendriks et al [63,64], provided valuable discussions and evaluations of impact.

Where papers reported on the impact of their involvement work, the results were largely positive. The studies demonstrated that PPI can have a positive impact on research quality at multiple stages of the research cycle as well as on those taking part. To achieve this, the authors reflected on the need to involve multiple stakeholder groups and use multiple methods of involvement to provide a person-centered and flexible approach in which people feel well supported and valued for their contribution. To do this evidently requires significant investment of time and resources. Even those papers detailing what we considered to be relatively high levels of involvement spoke about the need for more time and resources, for example, to improve levels of training or offer more formalized PPI roles [51,69].

It was helpful to see negative experiences with PPI also reported [56,63,64]. These are often missing from the literature [85] but provide helpful learning points. Another area that received little attention is the emotional impact of PPI on researchers. This was commented on briefly by Hendriks et al [64] but otherwise did not feature in the studies we reviewed, although it has been noted as a feature of PPI in dementia research more broadly [43,84]. The emotional impact on researchers might be seen as both positive and negative—as a research community, it would be helpful to recognize and value the learning and increased empathy that can come from close working with patient and public partners. It is also important to recognize the potential distress or emotional burden felt by researchers, which might be associated with this relationship, in particular for those unused to working with people in cognitive decline [43]. It is important that future studies consider these impacts and that researchers as well as patient and public partners have access to adequate training and support.

Across most studies (27/31, 87%), formal or standardized methods were not used to capture impact. Reporting was generally limited to the authors’ personal reflections. In only 13% (4/31) of the papers did the authors report seeking direct feedback from those who had been involved, for example, in the form of interviews or evaluation forms [51,56,63,73]. Hendriks et al [63] included a detailed evaluation of impact. This team retrospectively analyzed their participatory design process, mapping out the decision-making process at each stage of the project and considering the extent to which coresearchers had been involved and, therefore, whether participation had been truly meaningful. They also interviewed some of those who had been involved and analyzed themes that emerged. There is a risk that reporting on the impact of PPI can lead to an overvaluing of that which is easily measured but of little meaning (such as the number of people involved) instead of these more complex issues such as research culture or power relations [85]. Therefore, this example by Hendriks et al [63] reflecting on and evaluating the power dynamics at play and the processes that took place is particularly commendable.

In the future, it would be helpful for all involved—researchers, patients, and the public—if there were more recording and reporting of the impact of involvement to help all parties understand if, when, how, and why partnership working is beneficial [20,43]. Capturing this in a meaningful way is the challenge ahead of us [85,86]. A focus on the dialogue and the learning is felt to be helpful—Russell et al [85] recommend exploring “the complexity and richness of this relationship, using methods that emphasise illumination rather than measurement, and asking when, why, and with whom the dialogue happens or fails to happen.”

### Defining PPI: Challenges We Faced in This Review

One of the challenges we faced was the varying terminology and approaches used to involve groups in research. We applied a broad definition of “involvement” and, therefore, included papers using co-design or participatory design processes, as described, for example, by Hendriks et al [63]. Within a participatory design approach, there is a deliberate blurring of the roles of “designer” and “end user.” When written up in a research context, this can lead to a blurring of the roles of researcher, designer, end user, and research participant. From a PPI perspective, it is not usually considered appropriate for people involved in research to also be research participants as this can compromise both the researcher and person involved [20]. However, the NIHR gives the example of participatory or action research as a possible exception to this rule, and it was often these types of studies that we reviewed. Nevertheless, it was often difficult to determine which studies met our inclusion criteria, in part because of this mixing of roles and because of lack of detail in the methodology sections. Where studies explicitly used qualitative research methods (stating a qualitative approach and collecting data for analysis with appropriate ethics approval), they were excluded from this review. However, details were often missing, or sometimes subsections of a study appeared to be qualitative, whereas other sections were framed more like PPI activities. Terminology could not be relied on as it was applied inconsistently among studies. Similarly, information about ethics applications was not always available, and we did not use this as part of the inclusion and exclusion criteria. Had we used a stricter definition of PPI, we would have excluded a significant portion of the literature (21/31, 68% of the studies in this review). We felt that doing this would result in a misrepresentation of the type of involvement work being carried out and in missing key learning points from these studies.

### Strengths

We used a comprehensive search strategy considering all types of technology, all types of dementia, and many terms for “patient and public involvement” to reflect the different types of involvement in the field, building on search strategies from other reviews [11,19,25,41,42] that at times had been narrower in scope (eg, looking at “patient and public involvement” but not “co-design”). The 2 reviewers overcame the difficulty of defining PPI through regular communication and close working throughout the screening stages, consulting with a third member of the team where required. The review is further strengthened by multidisciplinary team input, with representatives from health care and health sciences as well as from design and technology backgrounds, which we hope ensures that our reflections and conclusions are of interest and applicable to a wide range of disciplines.

### Limitations

We did not conduct a gray literature search. Doing so might have resulted in a broader range of accounts of involvement being included. In addition, we did not involve patients or the public in this review, which may have contributed additional perspective and depth. However, we have planned and started recruitment for a much broader PPI strategy for our research center. The results of this review will be shared with our steering group so as to jointly consider how the findings should inform our PPI work as a center.

### Conclusions

At present, most involvement in technology-related dementia research is limited in breadth (often to just 1 stage in the research cycle) and depth (often consultative rather than with any sharing of power). We see across the literature shared aspirations of high levels of meaningful involvement in research, and it is encouraging to see some evidence of this being put into practice, with some reporting on methods used for involvement and the impact this has. Where papers gave details, it appears that a flexible approach with multiple methods used at different stages of the research cycle may be the most successful, tailoring methods to the various groups or individuals involved and facilitating greater depth or breadth of involvement according to people’s wishes and abilities. When this is done well, PPI can have a positive impact on both the research and those involved. This evidently will take significant time and resources, particularly if the approaches used are to move beyond consultations to collaboration or coproduction. Wider reporting of methods and facilitative strategies along with more formalized methods for recording and reporting on meaningful impact would be helpful so that all those involved—researchers, patients, and other stakeholders—can understand and learn how best to jointly conduct research.

## Appendix

Multimedia Appendix 1 Detailed search strings used for the different databases.

Multimedia Appendix 2 Stakeholder groups included as part of patient and public involvement activities.

Multimedia Appendix 3 PRISMA-ScR (Preferred Reporting Items for Systematic Reviews and Meta-Analyses Extension for Scoping Reviews) checklist.

## Abbreviations

NIHR: National Institute for Health and Care Research
PPI: patient and public involvement
PRISMA-ScR: Preferred Reporting Items for Systematic Reviews and Meta-Analyses Extension for Scoping Reviews
