# Supplementary material for: Patient and Public Involvement in Technology-Related Dementia Research: Scoping Review
Source: JMIR Aging. 2024 Mar 4;7:e48292. doi: 10.2196/48292 (PMC10949132; doi:10.2196/48292)
Supplement: Multimedia Appendix 1 [file aging_v7i1e48292_app1.docx]

### Patient and Public Involvement in technology-related dementia research: a scoping review

# Multimedia Appendix: Search Strings

**Medline Search String (using Ovid)**

1. Dementi*.tw,kw.
2. Alzheimer*.tw,kw.
3. Lewy bod*.tw,kw.
4. Dementia/
5. Lewy Body Disease/
6. Dementia, Vascular/
7. Frontotemporal Dementia/
8. Dementia, Multi-Infarct/
9. ‘Mild cognitive impairment’.tw,kw.
10. MCI.tw,kw.
11. Parkinson Disease/
12. Aphasia, primary progressive/
13. Or/1-12
14. Public involv*.tw,kw.
15. Patient* involv*.tw,kw.
16. ‘Patient* and public involv*’.tw,kw.
17. Community-Based Participatory Research/
18. James Lind Alliance.tw,kw.
19. ((patient* or consumer* or stakeholder* or user* or lay* or carer* or public or client* or citizen* or communit* or caregiver* or family or families or resident*) adj1 (involve* or consult* or contribut* or engage* or activat* or advis* or opinion* or dialog* or voice* or partner* or input* or participat* or collaborat* or panel or empower* )).kw,tw.
20. (co-research* or coresearch*).tw,kw.
21. (co-design* or co-design*).tw,kw.
22. ((participatory or user-cent* or human-cent*) adj2 (design* or research*)).tw,kw.
23. Patient Participation/
24. Community Participation/
25. Or/14-24
26. Biomedical Technology/
27. Telehealth.tw.
28. Telemedicine/
29. Artificial intelligence/
30. Artificial intelligence.tw.
31. Machine learning.tw.
32. Machine Learning/
33. Technolog*.tw.
34. Digital*.tw.
35. ((assistive or smart or home*) adj2 (technolog* or device* or system*)).tw.
36. Monitor*.tw
37. Virtual.tw
38. Or/26-35
39. 13 and 25 and 36
40. Limit 37 to English

**EMBASE search string (using Ovid)**

1. Dementi*.tw,kw.
2. Alzheimer*.tw,kw.
3. Lewy bod*.tw,kw.
4. Dementia/
5. Lewy Body Disease/
6. Dementia, Vascular/
7. Frontotemporal Dementia/
8. Dementia, Multi-Infarct/
9. ‘Mild cognitive impairment’.tw,kw.
10. MCI.tw,kw.
11. Parkinson Disease/
12. Aphasia, primary progressive/
13. Or/1-12
14. Public involv*.tw,kw.
15. Patient* involv*.tw,kw.
16. ‘Patient* and public involv*’.tw,kw.
17. Community-Based Participatory Research/
18. James Lind Alliance.tw,kw.
19. ((patient* or consumer* or stakeholder* or user* or lay* or carer* or public or client* or citizen* or communit* or caregiver* or family or families or resident*) adj1 (involve* or consult* or contribut* or engage* or activat* or advis* or opinion* or dialog* or voice* or partner* or input* or participat* or collaborat* or panel or empower* )).kw,tw.
20. (co-research* or coresearch*).tw,kw.
21. (co-design* or co-design*).tw,kw.
22. ((participatory or user-cent* or human-cent*) adj2 (design* or research*)).tw,kw.
23. Patient Participation/
24. Community Participation/
25. Or/14-24
26. Biomedical Technology/
27. Telehealth.tw.
28. Telemedicine/
29. Artificial intelligence/
30. Artificial intelligence.tw.
31. Machine learning.tw.
32. Machine Learning/
33. Technolog*.tw.
34. Digital*.tw.
35. ((assistive or smart or home*) adj2 (technolog* or device* or system*)).tw.
36. Monitor*.tw
37. Virtual.tw
38. Or/26-35
39. 13 and 25 and 36
40. Limit 37 to English

**CINAHL search string (using EBSCO)**

1. (MH “Dementia”) OR (MH “Frontotemporal Dementia”) OR (MH “Dementia, Vascular”) OR (MH “Dementia, Multi-Infarct”) OR (“MH “Lewy Body Disease”)
2. (MH “Alzheimer’s Disease”)
3. TI dementia* OR AB dementia*
4. AB Alzheimer* OR TI Alzheimer*
5. TI lewy bod* OR AB lewy bod*
6. AB ( mild cognitive impairment or mci ) OR TI ( mild cognitive impairment or mci )
7. (MH “Parkinson Disease”)
8. AB primary progressive aphasia or ppa
9. TI primary progressive aphasia or ppa
10. S1 OR S2 OR S3 OR S4 OR S5 OR S6 OR S7 OR S8 OR S9
11. AB public involv*
12. TI public involv*
13. AB patient* involv*
14. TI patient* involv*
15. AB “patient* and public involv*”
16. TI “patient* and public involv*”
17. AB James Lind Alliance
18. AB ((patient* or consumer* or stakeholder* or user* or lay* or carer* or public or client* or citizen* or communit* or caregiver* or family or families or resident*) N1 (involve* or consult* or contribut* or engage* or activat* or advis* or opinion* or dialog* or voice* or partner* or input* or participat* or collaborat* or panel or empower* ))
19. TI ((patient* or consumer* or stakeholder* or user* or lay* or carer* or public or client* or citizen* or communit* or caregiver* or family or families or resident*) N1 (involve* or consult* or contribut* or engage* or activat* or advis* or opinion* or dialog* or voice* or partner* or input* or participat* or collaborat* or panel or empower* ))
20. AB (co-research* or coresearch*)
21. TI (co-research* or coresearch*)
22. AB ( (co-design* or codesign*) ) OR TI ( (co-design* or codesign*) )
23. AB ( ((participatory or user-cent* or human-cent*) N1 (design* or research*)) ) OR TI ( ((participatory or user-cent* or human-cent*) N1 (design* or research*)) )
24. (MH "Consumer Participation")
25. S11 OR S12 OR S13 OR S14 OR S15 OR S16 OR S17 OR S18 OR S19 OR S20 OR S21 OR S22 OR S23 OR S24
26. (MH "Digital Technology") OR (MH "Assistive Technology")
27. (MH "Telehealth")
28. (MH "Telemedicine") OR (MH "Telerehabilitation")
29. (MH "Artificial Intelligence")
30. (MH "Machine Learning")
31. TI artificial intelligence OR AB artificial intelligence
32. TI machine learning OR AB machine learning
33. TI telehealth OR AB telehealth
34. TI technolog* OR AB technolog*
35. TI digital* OR AB digital*
36. TI ( ((assistive or smart or home*) N1 (technolog* or device* or system*)) ) OR AB ( ((assistive or smart or home*) N1 (technolog* or device* or system*)) )
37. AB monitor* OR TI monitor*
38. TI virtual OR AB virtual
39. S26 OR S27 OR S28 OR S29 OR S30 OR S31 OR S32 OR S33 OR S34 OR S35 OR S36 OR S37 OR S38
40. S10 AND S25 AND S39
41. LA English
42. S40 AND S41

**APA PsycInfo search string (using Ovid)**

1. Dementi*.tw,kw.
2. Alzheimer*.tw,kw.
3. Lewy bod*.tw,kw.
4. ‘Mild cognitive impairment’.tw,kw.
5. MCI.tw,kw
6. ‘Parkinson*disease’.tw,kw.
7. 1 or 2 or 3 or 4 or 5 or 6
8. Public involve*.tw,kw
9. Patient* involv*.tw,kw.
10. Patient* and public involv*.tw,kw
11. Community based participatory research.tw,kw
12. James Lind Alliance.tw,kw
13. ((patient* or consumer* or stakeholder* or user* or lay* or carer* or public or client* or citizen* or communit* or caregiver* or family or families or resident*) adj1 (involve* or consult* or contribut* or engage* or activat* or advis* or opinion* or dialog* or voice* or partner* or input* or participat* or collaborat* or panel or empower* )).kw,tw.
14. (co-research* or coresearch*).tw,kw.
15. (co-design* or co-design*).tw,kw.
16. ((participatory or user-cent* or human-cent*) adj2 (design* or research*)).tw,kw.
17. 8 or 9 or 10 or 11 or 12 or 13 or 14 or 15 or 16
18. Telehealth.tw,kw
19. Biomedical technology.tw,kw
20. Telemedicine.tw,kw
21. Artificial intelligence.tw,kw
22. Machine learning.tw,kw
23. Technolog*.tw,kw.
24. Digital*.tw,kw.
25. ((assistive or smart or home*) adj2 (technolog* or device* or system*)).tw.kw.
26. Monitor*.tw,kw.
27. Virtual.tw,kw.
28. 18 or 19 or 20 or 21 or 22 or 23 or 24 or 25 or 26 or 27
29. 7 and 17 and 28
