# Supplementary figures and images for: Patient and Public Involvement in Technology-Related Dementia Research: Scoping Review
Source: JMIR Aging. 2024 Mar 4;7:e48292. doi: 10.2196/48292 (PMC10949132; doi:10.2196/48292)

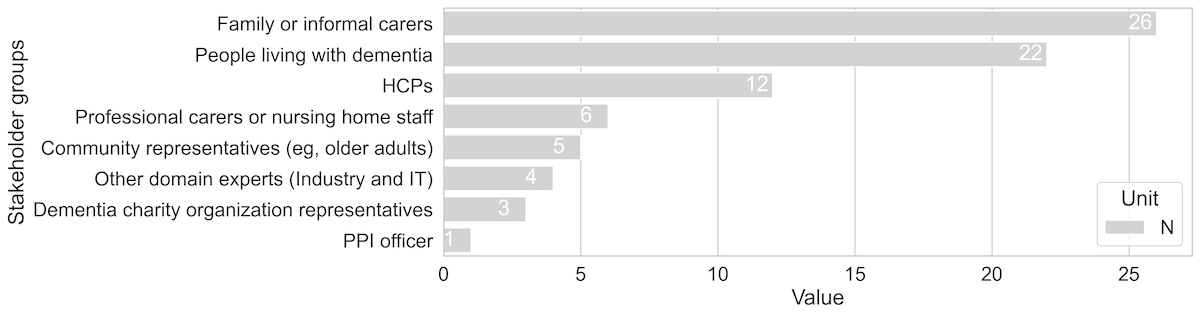

Supplement: Multimedia Appendix 2 [file aging_v7i1e48292_app2.png]
